# Prognostische Einflussfaktoren auf den Erfolg der Sialendoskopie bei Sialolithiasis

**DOI:** 10.1055/a-1510-9548

**Published:** 2021-05-31

**Authors:** Aris I Giotakis, Rene Fischlechner, Daniel Dejaco, Timo Gottfried, Herbert Riechelmann

**Affiliations:** Universitätsklinik für Hals-, Nasen- und Ohrenheilkunde, Medizinische Universität Innsbruck, Austria

**Keywords:** Speicheldrüsen, Sialolithiasis, Endoskopie, Prognose, Sialendoskopie, LSD-Klassifikation, salivary glands, salivary gland calculi, endoscopy, prognosis, sialendoscopy, LSD classification

## Abstract

**Hintergrund** Der Erfolg der Sialendoskopie hängt von mehreren Faktoren ab. Im Jahr 2008 wurde die Lithiasis-Stenosis-Dilatation (LSD) -Klassifikation zur genaueren Beschreibung des Stein-Gang-Verhältnisses eingeführt. Neben dem Nutzen der LSD-Klassifikation untersuchten wir weitere prä- und intraoperativ erhobene Einflussfaktoren auf den Erfolg der Sialendoskopie bei Sialolithiasis.

**Methode** PatientInnen mit Sialolithiasis der Glandula submandibularis und Glandula parotis, die zwischen September 2018 und März 2020 eine primäre Sialendoskopie erhielten, wurden retrospektiv untersucht. Die Steingröße, Steinlokalisation und LSD-Klassifikation wurden unter anderem als prognostische Einflussfaktoren untersucht.

**Ergebnisse** Insgesamt wurden 37 PatientInnen retrospektiv untersucht. Eine erfolgreiche Steinextraktion wurde bei 12/37 (32 %) PatientInnen durchgeführt. Bei Erfolg betrug die mediane Steingröße 3,7 mm, verglichen mit 10 mm bei Misserfolg (Mann-Whitney-Test; p < 0,0001). Bei Erfolg waren 11/12 Steine distal lokalisiert, verglichen mit 13/25 proximal lokalisierten Steinen bei Misserfolg (Pearson-Chi-Quadrat-Test; p = 0,010). Bei Erfolg wurden 10/12 Steine als L1S0D0 klassifiziert, während 15/25 bei Misserfolg als L3aS0D0-Steine klassifiziert wurden (Pearson-Chi-Quadrat-Test; p = 0,001). Für distal lokalisierte Steine, die kleiner als 5 mm waren, lag die Erfolgsrate bei 100 %. Für proximal lokalisierte Steine, die größer als 4 mm waren, lag die Erfolgsrate bei 0 %. Für die als L1S0D0 klassifizierten Steine betrug die Erfolgsrate 60–100 %.

**Schlussfolgerung** Distal lokalisierte Steine und Steine kleiner als 5 mm in einem ansonsten unauffälligen Gangsystem können als prognostisch günstige Faktoren angesehen werden. Zukünftige Studien sollten anhand größerer Datenmengen die LSD-Klassifikation, das Volumen der Steine und deren Gangorientierung bzw. deren Abstand von der Papille untersuchen.

## Abstract (en)

**Background** The success of sialendoscopy depends on several factors. The 2008 introduced lithiasis-stenosis-dilatation (LSD) classification intended to describe more precise the stone-duct system. We investigated whether the LSD classification and additional pre- and intraoperative parameters could be used as prognostic factors for success.

**Methods** We retrospectively assessed patients with primary sialendoscopy for sialolithiasis between September 2018 und March 2020. Among others, the outcome variables were the stone size, location and LSD classification.

**Results** We included 37 patients. The success group included 12/37 (32 %) patients. The median stone size was 3.7 millimeters (mm) in the success group and 10.0 mm in the failure group (Mann-Whitney test; p < 0.0001). In the success group, 11/12 stones were distal in contrast to the failure group (13/25 stones proximal; Pearson's chi-square test; p = 0.010). We noted 10 L1S0D0 stones in the success group in contrast to the failure group (15 L3aS0D0 stones; Pearson chi-square test; p = 0.001). For distal stones smaller than 5 mm, the success rate was 100 % and for proximal stones larger than 4 mm, it was 0 %. For stones free in the duct lumen (L1S0D0), the success rate was 60–100 %.

**Conclusion** The distal stone location and the smaller stone size in a normal duct should be beneficial prognostic factors for success. Future studies should focus on the LSD classification, stone volume, stone duct orientation and stone distance from the papilla.

## Einleitung

Die Sialendoskopie ist definiert als Endoskopie der Speicheldrüsengänge über ihr natürliches Ostium. Diese Technik wurde 1990 erstmals von 2 separaten Gruppen gleichzeitig beschrieben 1 2 . Konigsberger und Co-Autoren berichteten von einer intraduktalen, laserinduzierten Lithotripsie bei einem Patienten mit rezidivierender, eitriger Sialadenitis der Glandula submandibularis 1 . Gundlach und Co-Autoren beschrieben eine Fallserie von 12 Patienten mit Sialolithiasis, bei denen eine intraduktale, laserinduzierte Stoßwellenlithotripsie unter kontinuierlicher Sialendoskopie durchgeführt wurde 2 . In den folgenden Jahren wurden die ersten sialendoskopisch kontrollierten Konkremententfernungen durchgeführt 3 4 5 6 7 . Die Sialendoskopie gilt aktuell als Referenzmethode zur Diagnose und Therapie obstruktiver Speicheldrüsenpathologien 8 .

Marchal und Co-Autoren berichteten, dass eine diagnostische Sialendoskopie in 98 % der Fälle möglich war 9 . Für die interventionelle Sialendoskopie wird die Erfolgsrate als deutlich niedriger beschrieben. Als prognostische Einflussfaktoren auf die Erfolgsrate der interventionellen Sialendoskopie wurden die Größe, Lokalisation, Orientierung und Mobilität des Konkrements diskutiert 8 10 11 12 13 . Eine Steingröße von ≤ 4 mm für submandibuläre Konkremente und eine von ≤ 3 mm für parotideale Konkremente wurde von Marchal und Co-Autoren als Schwellenwert für eine erfolgreiche, sialendoskopische Steinextraktion berechnet 8 . Unter Achtung dieser Schwellenwerte wurde eine Erfolgsrate von 80–89 % beschrieben 11 12 . Cox und Co-Autoren beschrieben als ergänzenden Einflussfaktor auf eine erfolgreiche sialendoskopische Steinextraktion einen Abstand des Konkrements zur Papille von ≤ 3 cm 13 .

Im Jahr 2008 wurde die Lithiasis-Stenosis-Dilatation (LSD) -Klassifikation von Marchal und Co-Autoren eingeführt 14 . Ziel der LSD-Klassifikation war die Standardisierung der Beschreibung der intraoperativen Befunde des Stein-Gang-Verhältnisses ( Tab. 1 ). „L“ steht für Lithiasis und beschreibt in 6 Subklassen die Größe des Konkrements, dessen Beziehung zum Gang und dessen Tastbefund. „S“ steht für Stenosis und beschreibt in 5 Subklassen den Schweregrad der Stenose des Gangs und deren Ausprägung im Gangsystem. „D“ steht für Dilatation und beschreibt in 4 Subklassen das Ausmaß der Gangerweiterung und deren Ausprägung im Gangsystem. Aktuell sind vorhandene Daten zum prognostischen Nutzen der LSD-Klassifikation auf den Erfolg einer interventionellen Sialendoskopie bei Sialolithiasis spärlich.

**Tab. 1:** Beschreibung der LSD-Klassifikation.

*Score*	sialendoskopischer Befund
**„L“ (Lithiasis)**	
*L0*	Gang frei von Steinen
*L1*	im Ganglumen freier Stein
*L2a*	festsitzender Stein, komplett einsehbar < 8 mm
*L2b*	festsitzender Stein, komplett einsehbar > 8 mm
*L3a*	festsitzender Stein, teilweise einsehbar, tastbar
*L3b*	festsitzender Stein, teilweise einsehbar, nicht tastbar
**„S“ (Stenose)**	
*S0*	keine Stenose
*S1*	intraduktale membranöse Stenose (einzige oder mehrere)
*S2*	einzige duktale Stenose vom Hauptgang
*S3*	multiple oder diffuse duktale Stenose vom Hauptgang
*S4*	generalisierte duktale Stenose
**„D“ (Dilatation)**	
*D0*	keine Dilatation
*D1*	einzige Dilatation
*D2*	multiple Dilatationen
*D3*	generalisierte Dilatation

In dieser retrospektiven Studie explorierten wir die LSD-Klassifikation und zusätzliche Parameter als mögliche prognostische Einflussfaktoren auf den Erfolg der interventionellen Sialendoskopie bei Sialolithiasis. Die untersuchten zusätzlichen Einflussfaktoren waren unter anderem die Steingröße, Steinlokalisation und Speicheldrüsenlokalisation. Ergänzend untersuchten wir, ob die Anzahl an sonografisch diagnostizierten Konkrementen mit der Anzahl der sialendoskopisch gesehenen Konkremente korreliert bzw. übereinstimmt.

## Material und Methoden

### Studiendesign

Patienten aus der „Sialendoskopie-Datenbank“ der Universitätsklinik für Hals-, Nasen- und Ohrenheilkunde, Kopf- und Halschirurgie wurden retrospektiv untersucht. In der „Sialendoskopie-Datenbank“ wurden prä-, intra- und postoperative Informationen seit September 2018 prospektiv gesammelt. Eingeschlossen wurden alle Patienten, die bei Sialolithiasis der Glandula submandibularis oder Glandula parotis eine primäre Sialendoskopie zwischen September 2018 und März 2020 erhielten. Patienten mit Revisions-Sialendoskopie wurden exkludiert. Alle Verfahren, die in dieser retrospektiven Studie durchgeführt wurden, entsprachen den ethischen Standards der Deklaration von Helsinki 1964 und deren späteren Revisionen.

### Definition des Erfolgs

Eine erfolgreiche sialendoskopische Steinextraktion wurde definiert als a) Extraktion mit und ohne Papillotomie für die Glandula submandibularis und b) ohne Papillotomie für die Glandula parotis, wie von Cox und Kollegen beschrieben 13 . Die erfolgreichen Eingriffe wurden in die „Erfolgsgruppe“ und die erfolglosen Eingriffe in die „Misserfolgsgruppe“ inkludiert.

### Outcome-Variablen

#### Vergleich der prä- und intraoperativen Parameter

Wir untersuchten, ob es bei den prä- und intraoperativen Parametern signifikante Unterschiede zwischen der Erfolgsgruppe und der Misserfolgsgruppe gab. Die untersuchten prä- und intraoperativen Parameter waren: 1) Geschlecht, 2) Alter, 3) Dauer der präoperativen Symptomatik, 4) betroffene Speicheldrüse, 5) Seite der betroffenen Speicheldrüse, 6) Steingröße, 7) Steinlokalisation (distal oder proximal), 8) Steinanzahl, 9) LSD-Klassifikation und 10) Operationsdauer. Ergänzend erfolgte die Untersuchung der Steingröße und der Steinlokalisation aufgeschlüsselt nach Speicheldrüse. Für die Steingröße wurde die Angabe der Halssonografie benutzt und die größte Dimension in mm dokumentiert. Die Halssonografien fanden in der Universitätsklinik für Radiologie innerhalb der letzten Wochen vor der Operation statt. Im Fall von mehreren Steinen wurden nur die Größe und Lokalisation des größeren Steins dokumentiert.

#### Prognose in Abhängigkeit von Steinparameter, Speicheldrüse und LSD-Klassifikation

Die Steingröße wurde in Clustern klassifiziert. Insgesamt wurden 9 Cluster gebildet, wobei das kleinste Cluster als Steine unter 3 mm und das größte Cluster als Steine über 10 mm definiert wurde. Zwischen 3 und 10 mm wurde pro mm je ein Cluster generiert. Die Erfolgsrate ermittelte sich als prozentualer Anteil der erfolgreichen Eingriffe zur Gesamtmenge der Eingriffe pro Cluster.

#### Korrelation und Übereinstimmung der Halssonografie und der Sialendoskopie

Abschließend untersuchten wir die Korrelation und Übereinstimmung der sonografisch diagnostizierten Steinanzahl (SONOsteine) und der sialendoskopisch gesehenen Steinanzahl (SIALsteine). Die Übereinstimmung wurde mit der Bland-Altman-Methode bestimmt 15 16 .

### Operationsausrüstung

Für alle Eingriffe wurden Produkte der Firma Bess (Berlin, Deutschland) benutzt. Die Standardausrüstung inkludierte: 1) eine semirigide noduläre Optik, Öffnungswinkel 70 ^o^ , Auflösung 6000 Pixel, Durchmesser 0,55 mm und Länge 181 mm, 2) 4-mal Polyshaft mit jeweils 0,60, 0,70, 1,05 und 1,40 cm Innendurchmesser und 90 mm Länge, 3) einen Handgriff, 4) einen Optik-Fixateur und 5) einen Heissner-Mundsperrer. Für Sondierung wurden 4 verschiedene Bowman-Sonden (00/0, 0/1, 1/2, 2/3 und 3/4) und verschiedene Dilatatoren verwendet. Die Interventionsausrüstung inkludierte: 1) ein 3-Draht-Steinfangkörbchen mit einem Fangkörbchenhandgriff, 2) einen Spiralbohrer, 3) einen Dilatationsballon mit einer Länge von 30 mm und 4) eine semirigide Fremdkörperzange.

### Operationsmethode

Alle Eingriffe wurden von 2 Operateuren in Vollnarkose durchgeführt. Nach Einführung des Heissner-Mundsperrers erfolgte für die Glandula submandibularis die Sondierung der Papille mittels Bowman-Sonden und/oder Dilatator. Kleinere Konkremente wurden mittels Fangkörbchen bzw. Fremdkörperzange entfernt. Bei Steinen größer als die Öffnung der Papille wurde eine Papillotomie durchgeführt. Bei größeren und fixierten Konkrementen wurde die Zertrümmerung mittels Spiralbohrer bzw. Fremdkörperzange versucht. Bei erfolgloser Zertrümmerung erfolgte eine Gangschlitzung. Für Eingriffe der Glandula parotis wurden kleine Konkremente mittels Fangkörbchen bzw. Fremdkörperzange entfernt. War der Stein größer als die Öffnung der Papille, wurde auch hier die Zertrümmerung mittels Spiralbohrer bzw. Fremdkörperzange versucht. Bei frustraner Zertrümmerung wurde der Eingriff abgebrochen und der Patient zur extrakorporalen Stoßwellenlithotripsie überwiesen. Beide Operateure haben vor September 2018 weniger als 10 Sialendoskopien durchgeführt.

### Statistik

Die Analyse wurde mit der Software IBM SPSS ^®^ Statistics 26 (SPSS Inc., Chicago, Illinois, Vereinigte Staaten) durchgeführt. Der Shapiro-Wilk-Test wurde für die Überprüfung der Normalverteilung benutzt. Für den Vergleich der kontinuierlichen unabhängigen normalverteilten Daten wurde der Student-T-Test und für den Vergleich der kategorialen Variablen der Pearson-Chi-Quadrat-Test verwendet. Der Mann-Whitney-Test wurde für nicht normalverteilte kontinuierliche unabhängige Variablen verwendet. Der Wilcoxon-Test wurde für nicht normalverteilte kontinuierliche verbundene Variablen benutzt. Für die Korrelation der kategorialen Variablen wurde der Pearson-Correlation-Test verwendet. Ein Ergebnis von p < 0,05 wurde als signifikant angesehen.

## Ergebnisse

### Prä- und intraoperative Variablen

Insgesamt wurden 37 Patienten retrospektiv untersucht, wovon 15 weiblich waren. Eine erfolgreiche Steinextraktion wurde bei 13/37 (32 %) durchgeführt. Eine Papillotomie wurde in 7/8 erfolgreichen Eingriffen der Glandula submandibularis benötigt. Das mittlere Alter ± die Standardabweichung (SD) betrug 48 ± 14 Jahre (21–81 Jahre) und war normalverteilt (p > 0,2). Weder Geschlecht noch Alter unterschieden sich signifikant zwischen der Erfolgs- und Misserfolgsgruppe (Pearson-Chi-Quadrat-Test; p = 0,13 bzw. Student-T-Test; p > 0,2; Tab. 2 ).

**Tab. 2:** Vergleich der prä- und intraoperativen Parameter zwischen der Erfolgs- und der Misserfolgsgruppe.

Parameter		Erfolgsgruppe (n = 12)	Misserfolgsgruppe (n = 25)	p-Wert
Alter (Jahre) a		47 ± 18	49 ± 12	> 0,2 b
Dauer der Symptomatik (Wochen) c		48 (7–48)	48 (34–180)	0,079 d
Operationsdauer (Minuten) c		80 (64–98)	106 (71–129)	0,098 d
SONOsteine c		1,0 (1,0–2,0)	1,0 (1,0–1,0)	> 0,2 d
SIALsteine c		1,0 (1,0–2,0)	1,0 (1,0–2,0)	0,093 d
Steingröße c (mm)		3,7 (3,0–5,0)	10,0 (5,3–13,0)	< 0,0001 d
Geschlecht	Frauen	7	8	0,13 e
	Männer	5	17	
Glandula	submandibularis	8	22	0,12 e
	parotis	4	3	
Seite	links	5	11	> 0,2 e
	rechts	7	14	
Lokalisation	distal	11	12	* 0,010 e*
	proximal	1	13	
LSD	L _1_ S _0_ D _0_	10	4	* 0,001 e*
	L _2a_ S _0_ D _0_	1	1	
	L _2b_ S _0_ D _0_	0	2	
	L _3a_ S _0_ D _0_	0	15	
	L _3b_ S _0_ D _0_	0	1	
	L _2a_ S _2_ D _0_	1	0	
	L _3a_ S _2_ D _0_	0	2	

a Mittelwert ± Standardabweichung.

b Student-T-Test.

c Medianwert (unteres Quartil bis oberes Quartil).

d Mann-Whitney-Test.

e Pearson-Chi-Quadrat-Test.

Die mediane Dauer der präoperativen Symptomatik betrug 48 Wochen (unteres Quartil 22 Wochen bis oberes Quartil 96 Wochen; 1–1680 Wochen) und war nicht normalverteilt (p < 0,0001). Die Dauer der präoperativen Symptomatik unterschied sich nicht signifikant zwischen den beiden Gruppen (Mann-Whitney-Test; p = 0,079; Tab. 2 ).

Der Eingriff erfolgte häufiger an der Glandula submandibularis (30/37) als an der Glandula parotis (7/37) und wurde in einem ähnlichen Seitenverhältnis durchgeführt. Weder die betroffene Speicheldrüse noch die Seite der Speicheldrüse unterschieden sich signifikant zwischen der Erfolgs- und Misserfolgsgruppe (Pearson-Chi-Quadrat-Test; p = 0,12 bzw. p > 0,2; Tab. 2 ).

Eine alleinige Halssonografie als präoperative Bildgebung wurde bei 29/37, eine alleinige Computertomografie (CT) bei 4/37 und beide Bildgebungen bei 4/37 Patienten durchgeführt. In den insgesamt 33/37 durchgeführten präoperativen Halssonografien wurden im Median 1,0 Konkrement (unteres Quartil 1,0 bis oberes Quartil 1,0; 0–3 Konkremente) -SONOsteine diagnostiziert. Die Anzahl der SONOsteine unterschied sich nicht signifikant zwischen der Erfolgs- und der Misserfolgsgruppe (Mann-Whitney-Test; p > 0,2; Tab. 2 ).

Die mediane Operationsdauer betrug 90 Minuten (unteres Quartil 67 bis oberes Quartil 118 Minuten; 20–270 Minuten) und war nicht normalverteilt (p = 0,003). Die Operationsdauer unterschied sich nicht signifikant zwischen der Erfolgs- und der Misserfolgsgruppe (Mann-Whitney-Test; p = 0,098; Tab. 2 ).

#### Steinparameter

Die mediane Steingröße betrug 5,5 mm (unteres Quartil 4,0 bis oberes Quartil 12mm; 2–22 mm) und war nicht normalverteilt (p = 0,002). Die Steingröße unterschied sich signifikant zwischen der Erfolgs- und der Misserfolgsgruppe. Sie war 3,7 mm (unteres Quartil 3,0 bis oberes Quartil 5,0mm; 2–5 mm) in der Erfolgsgruppe und 10,0 mm (unteres Quartil 5,3 bis oberes Quartil 13,0mm; 4–22 mm) in der Misserfolgsgruppe (Mann-Whitney-Test; p < 0,0001; Tab. 2 ). Die Steingröße korrelierte nicht mit der Dauer der präoperativen Symptomatik (p > 0,2).

Von den 37 Konkrementen waren 23 distal und 14 proximal lokalisiert. Die Steinlokalisation unterschied sich signifikant zwischen der Erfolgs- und der Misserfolgsgruppe. In der Erfolgsgruppe dokumentierten wir 11 distale Steine und einen proximalen Stein. Die Misserfolgsgruppe inkludierte 12 distale und 13 proximale Steine (Pearson-Chi-Quadrat-Test; p = 0,010).

Die intraoperativ beschriebenen Steine wurden am häufigsten als L _1_ S _0_ D _0_ (14/37) oder als L _3a_ S _0_ D _0_ (15/37) klassifiziert. Die LSD-Klassifikation unterschied sich signifikant zwischen der Erfolgs- und der Misserfolgsgruppe. In der Erfolgsgruppe gab es 10 L _1_ S _0_ D _0_ -Steine und keine L _3a_ S _0_ D _0_ -Steine; in der Misserfolgsgruppe gab es 4 L _1_ S _0_ D _0_ -Steine und 15 L _3a_ S _0_ D _0_ -Steine (Pearson-Chi-Quadrat-Test; p = 0,001). Die meisten L _1_ S _0_ D _0_ -Steine lagen distal (13/14). Die L _3a_ S _0_ D _0_ -Steine lagen entweder distal (6/15) oder proximal (9/15). Der Unterschied zwischen der Lokalisation der L _1_ S _0_ D _0_ -Steine und der Lokalisation der L _3a_ S _0_ D _0_ -Steine war signifikant (Pearson-Chi-Quadrat-Test; p = 0,016).

Die mediane Anzahl der SIALsteine war 1,0 (unteres Quartil 1,0 bis oberes Quartil 1,5 Konkremente; 1–3 Konkremente). Die Anzahl der SIALsteine unterschied sich nicht signifikant zwischen der Erfolgsgruppe und der Misserfolgsgruppe (Mann-Whitney-Test; p = 0,093; Tab. 2 ).

#### Speicheldrüse

Bei den 30 durchgeführten Sialendoskopien an der Glandula submandibularis betrug die mediane Steingröße 7,5 mm (unteres Quartil 4,9 bis oberes Quartil 12,3mm; 2–22 mm). Die Steingröße unterschied sich signifikant zwischen der Erfolgs- und der Misserfolgsgruppe. In der Erfolgsgruppe betrug die Steingröße 3,7 mm (unteres Quartil 3,0 bis oberes Quartil 5,0mm; 2–5 mm), in der Misserfolgsgruppe betrug sie 10,5 mm (unteres Quartil 6,0 bis oberes Quartil 13,0mm; 4–22mm; Mann-Whitney-Test; p = 0,001).

Die Steinlokalisation bei den Eingriffen an der Glandula submandibularis unterschied sich signifikant zwischen der Erfolgs- und der Misserfolgsgruppe (Pearson-Chi-Quadrat-Test; p = 0,040). In der Erfolgsgruppe gab es 7 distale Steine und einen proximalen Stein. In der Misserfolgsgruppe gab es 10 distale Steine und 12 proximale Steine.

**Abb. 1:**
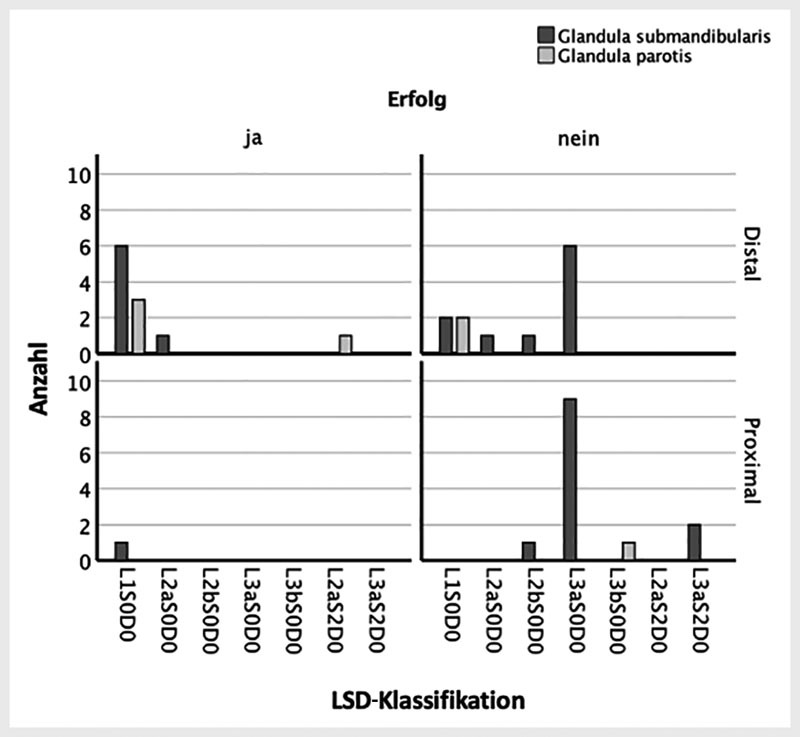
Vergleich der LSD-Klassifikation zwischen der Erfolgs- und der Misserfolgsgruppe. Y-Achse: Anzahl der Steine mit der jeweiligen LSD-Klassifikation. X-Achse: LSD-Klassifikation. Mit Dunkelgrau wird die Glandula submandibularis und mit Hellgrau die Glandula parotis beschrieben. Die oberen Grafiken entsprechen den distalen Steinen und die unteren Grafiken den proximalen Steinen. Die linken Grafiken entsprechen der Erfolgsgruppe und die rechten Grafiken der Misserfolgsgruppe.

Die LSD-Klassifikation bei den Eingriffen an der Glandula submandibularis unterschied sich signifikant zwischen der Erfolgs- und der Misserfolgsgruppe. Die Mehrheit der Steine gehörte wieder der L _1_ S _0_ D _0_ - und L _3a_ S _0_ D _0_ -Klassifikation an. In der Erfolgsgruppe gab es 7 L _1_ S _0_ D _0_ - und keine L _3a_ S _0_ D _0_ -Steine. In der Misserfolgsgruppe gab es 2 L _1_ S _0_ D _0_ - und 15 L _3a_ S _0_ D _0_ -Steine (Pearson-Chi-Quadrat-Test; p = 0,0001; Abb. 1 ).

Bei den 7 Eingriffen an der Glandula parotis betrug die Steingröße 4,0 mm (unteres Quartil 3,0 bis oberes Quartil 5,0mm; 2–5,5 mm). Die Steingröße unterschied sich nicht signifikant zwischen der Erfolgs- und der Misserfolgsgruppe (Mann-Whitney-Test; p = 0,15). In der Erfolgsgruppe entsprach die Steingröße 3,5 ± 1,3 mm (2,0–5,0 mm), in der Misserfolgsgruppe war die Steingröße 5,0 ± 0,8 mm (4,0–5,5 mm).

Die Steinlokalisation bei den Eingriffen an der Glandula parotis unterschied sich nicht signifikant zwischen der Erfolgs- und der Misserfolgsgruppe (Pearson-Chi-Quadrat-Test; p > 0,2). In der Erfolgsgruppe gab es 4 distale und keine proximalen Steine. In der Misserfolgsgruppe gab es 2 distale Steine und einen proximalen Stein.

Bei Eingriffen an der Glandula parotis unterschied sich die LSD-Klassifikation nicht signifikant zwischen der Erfolgs- und der Misserfolgsgruppe. Hierzu gehörten die Steine klassifiziert als L _1_ S _0_ D _0_ , L _3b_ S _0_ D _0_ und L _2a_ S _2_ D _0_ . In der Erfolgsgruppe gab es 3 L _1_ S _0_ D _0_ - und einen L _2a_ S _2_ D _0_ -Stein. In der Misserfolgsgruppe gab es 2 L _1_ S _0_ D _0_ - und einen L _3b_ S _0_ D _0_ -Stein (Pearson-Chi-Quadrat-Test; p > 0,2; Abb. 1 ).

### Prognose in Abhängigkeit von Steinparametern, betroffener Speicheldrüse und LSD-Klassifikation

**Abb. 2:**
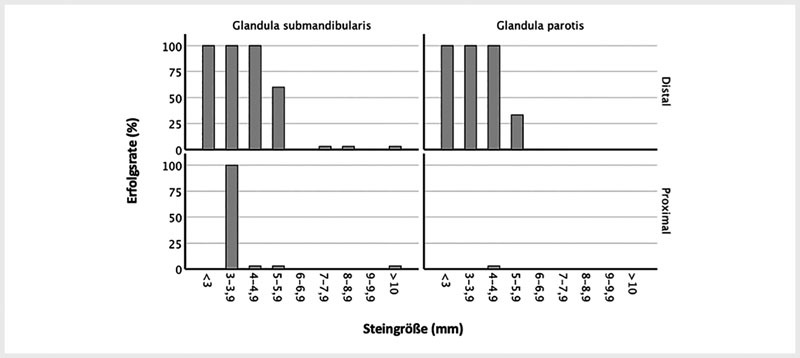
Erfolgsrate je nach Größe und Lokalisation der Steine, aufgeschlüsselt nach Speicheldrüse. Y-Achse: Prozentuale Erfolgsrate. X-Achse: Die Steingröße in mm. Die oberen Grafiken entsprechen den distalen Steinen und die unteren Grafiken den proximalen Steinen. Die linken Grafiken entsprechen Eingriffen an der Glandula submandibularis und die rechten Grafiken Eingriffen an der Glandula parotis. Die Säulen, die knapp über 0 sind, entsprechen einer 0 %-Erfolgsrate bei der jeweiligen Steingröße.

Für distale Steine kleiner als 5 mm lag die Erfolgsrate für beide Speicheldrüsen bei 100 % ( Abb. 2 ). Für proximale Steine größer als 4 mm lag die Erfolgsrate für beide Speicheldrüsen bei 0 % ( Abb. 2 ).

**Abb. 3:**
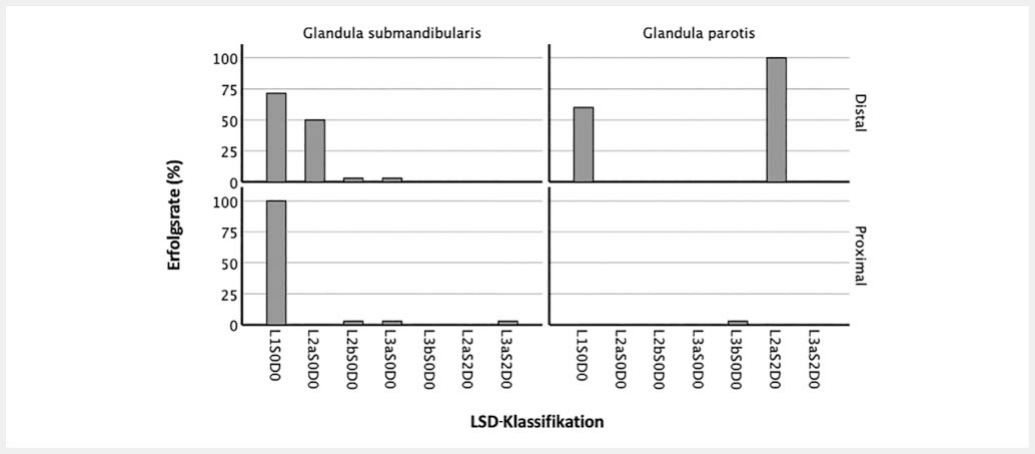
Erfolgsrate je nach LSD-Klassifikation, aufgeschlüsselt nach Speicheldrüse. Y-Achse: Prozentuale Erfolgsrate. X-Achse: LSD-Klassifikation. Die oberen Grafiken entsprechen den distalen Steinen und die unteren Grafiken den proximalen Steinen. Die linken Grafiken entsprechen Eingriffen an der Glandula submandibularis und die rechten Grafiken Eingriffen an der Glandula parotis. Die Säulen, die knapp über 0 sind, entsprechen einer 0 %-Erfolgsrate bei der jeweiligen Steingröße.

Für im Ganglumen frei flottierende Steine (L _1_ S _0_ D _0_ ) war die Erfolgsrate gleich oder über 60 %, unabhängig von der Steinlokalisation und der Speicheldrüse ( Abb. 3 ). Für größere, komplett einsehbare (L _2b_ S _0_ D _0_ ) oder teilweise einsehbare (L _3a_ S _0_ D _0_ oder L _3b_ S _0_ D _0_ ) Steine der Glandula submandibularis war die Erfolgsrate 0 % ( Abb. 3 ).

### Korrelation und Übereinstimmung der Anzahl der Steine der Halssonografie und der Sialendoskopie

Eine Halssonografie wurde bei 33/37 Patienten durchgeführt. Die Anzahl der SONOsteine korrelierte schwach mit der Anzahl der SIALsteine (r = 0,55; p = 0,001).

**Abb. 4:**
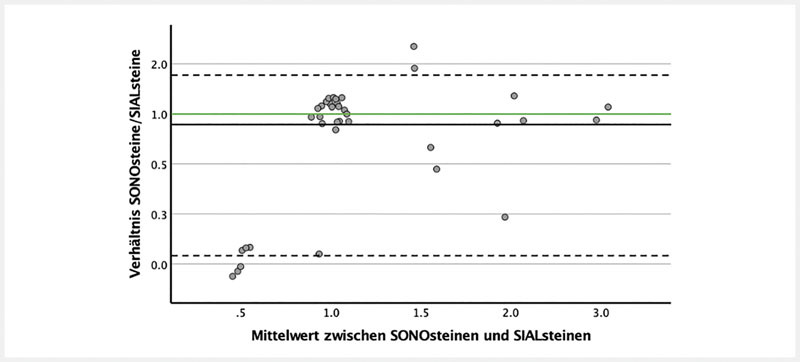
Übereinstimmung der Steinanzahl zwischen der Halssonografie und der Sialendoskopie bei der Bland-Altman-Methode mit Jitter-basierten Punkten. X-Achse: Mittelwert der Anzahl der SONOsteine und der Anzahl der SIALsteine. Y-Achse: Verhältnis SONOsteine/SIALsteine. Die grüne, horizontale Linie entspricht der absoluten Übereinstimmung zwischen beiden Methoden (SONOsteine = SIALsteine, d. h., SONOsteine/SIALsteine = 1). Die schwarze horizontale Linie entspricht dem Mittelwert (= 0,92) des Verhältnisses zwischen beiden Methoden (SONOsteine/SIALsteine). Die gestrichelten, horizontalen Linien entsprechen den 95 %-Konfidenzintervallen des Mittelwertes.

Dennoch konnten wir keinen signifikanten Unterschied zwischen der Anzahl der SONOsteine und der SIALsteine beobachten (Wilcoxon-Test basiert auf den negativen Ranken; p = 0,107). Der Mittelwert ± die Konfidenzintervalle des Verhältnisses zwischen SONOsteinen und SIALsteinen war 0,92 ± 0,84, d. h., die Anzahl der SIALsteine war 1,09 ± 1,19-mal höher als die Anzahl der SONOsteine ( Abb. 4 ).

## Diskussion

Im Jahr 2008 schlugen Marchal und Co-Autoren die LSD-Klassifikation für die Beurteilung intraduktaler Pathologien vor 14 . Die Autoren hofften, dass die LSD-Klassifikation insbesondere für multizentrische prospektive Studien nützlich sein könnte. Die LSD-Klassifikation hat den Vorteil, die intraduktalen Pathologien vollständig beurteilen zu können. Im Gegensatz zum Parameter „Steingröße“ wird bei der LSD-Klassifikation das Verhältnis des Steins zu dessen Umfeld besser betrachtet und das Stein-Gang-Verhältnis besser evaluiert. Vorhandene Daten zum prognostischen Nutzen der LSD-Klassifikation sind spärlich. Dementsprechend untersuchten wir in dieser retrospektiven Studie den Nutzen der LSD-Klassifikation als prognostischen Einflussfaktor auf die Erfolgsrate bei interventionellen Sialendoskopien für Sialolithiasis. Eine erfolgreiche sialendoskopische Steinextraktion wurde definiert als a) Extraktion mit und ohne Papillotomie für die Glandula submandibularis und b) ohne Papillotomie für die Glandula parotis.

Die vorliegenden zusammengefassten Daten für die Glandula submandibularis und Glandula parotis suggerieren, dass frei flottierende Steine in einem ansonsten Stenose- bzw. dilatationsfreien Gangsystem (L _1_ S _0_ D _0_ ) einfacher zu entfernen sind. In diesen Fällen lag die Erfolgsrate bei 60–100 %. Diese Beobachtung unterstreicht eine Limitation der LSD-Klassifikation. Die Lithiasis-Subklasse (L) subsumiert qualitative und quantitative Aspekte eines Konkrements in einem Parameter. Ein L _1_ -Konkrement wird unabhängig von der Steingröße als frei flottierend definiert. Die Steingröße selbst findet erst ab L _2_ Einfluss in die Klassifikation ( Tab. 1 ). Dementsprechend erklärt sich die Diskrepanz von einer 60–100 %-Erfolgsrate für frei flottierende Konkremente (also: L _1_ ) verglichen mit einer 100 %-Erfolgsrate für Steine ≤ 4 mm ( Abb. 2 ). L _1_ -Konkremente können demnach auch über 4 mm groß sein, was eine sialendoskopische Extraktion erschwert. Die Breite des Wharton-Gangs variiert zwischen 2 und 4 mm und die des Stenon-Gangs zwischen 1 und 2 mm 17 . Tatsächlich kann ein Konkrement von über 4 mm Größe aufgrund des durch die Spülung dilatierten Wharton-Gangs in diesem frei flottieren. Dass die höheren L-Subklassen im Wesentlichen mit der Steingröße korrelieren, spiegelt sich auch in unseren Beobachtungen wider: Die Erfolgsrate für höhere LSD-Klassifikationen war im Regelfall deutlich geringer.

Der Versuch der Datendokumentation dieser retrospektiven Studie deckte noch mehrere wesentliche Nachteile der LSD-Klassifikation als möglichen prognostischen Einflussfaktor auf den Erfolg der interventionellen Sialendoskopie bei Sialolithiasis auf. Diese Nachteile betreffen die fragliche Wichtigkeit des Tastbefundes und die fehlende Möglichkeit der Dokumentation der genauen Lokalisation des Konkrements. In der LSD-Klassifikation ist das Einsehen des Konkrements für die Unterscheidung zwischen L _2_ und L _3_ entscheidend. Marchal und Co-Autoren wählten diesen Parameter aus, da ein komplett einsehbares Konkrement (L _2_ ) im Gegensatz zu einem teilweise einsehbaren Konkrement (L _3_ ) leichter mit dem Laser fragmentiert werden kann 14 . Für die Unterscheidung zwischen L _3a_ und L _3b_ wurde der Tastbefund des Konkrements benutzt. Jedoch begründeten Marchal und Co-Autoren nicht, warum der Tastbefund in die LSD-Klassifikation inkludiert wurde und warum der Tastbefund für die sialendoskopische Extraktion eines Konkrements wichtig ist 14 . Der Tastbefund ist eher relevant für die chirurgische Steinextraktion durch die transorale Gangchirurgie der Glandula submandibularis oder seltener durch die transkutane Gangchirurgie der Glandula parotis. Außerdem ist die Lokalisation des Konkrements für die sialendoskopische Extraktion von großer Bedeutung. Die Literatur weist auf eine einfachere sialendoskopische Extraktion der distal gelegenen frei flottierenden Konkremente im Vergleich zu den proximal gelegenen frei flottierenden Konkrementen hin 13 . Beide Konkremente wären in dem Fall L _1_ S _0_ D _0_ -Konkremente laut der LSD-Klassifikation. Hier wird die Steinlokalisation als wichtiger Parameter des Erfolgs der Sialendoskopie bei Sialolithiasis nicht berücksichtigt. Ergänzend könnte man vermerken, dass die Klassifikation der Konkremente nach dem LSD-Schema zu einer unpraktischen Verkomplizierung führen könnte. Ein L _1_ S _0_ D _0_ -Konkrement könnte auch als freier, mobiler Stein bezeichnet werden.

Noch ein wesentlicher Nachteil der LSD-Klassifikation als prognostischer Einflussfaktor für den Erfolg einer Sialendoskopie entspricht der Tatsache, dass die LSD-Klassifikation erst intraoperativ bestimmt werden kann. Aus diesem Grund untersuchten wir ergänzend, welche anderen prä- und intraoperativen Parameter als prognostische Faktoren für den Erfolg einer Sialendoskopie bei Sialolithiasis dienen könnten. Die vorliegenden Daten suggerieren, dass insbesondere die Steingröße und die Steinlokalisation wesentliche prognostische Einflussfaktoren auf den Erfolg der Steinextraktion bei Sialendoskopie darstellen. Die höchste Erfolgsrate wurde bei kleinen Steinen, die distal lokalisiert waren, beobachtet. Diese Beobachtung deckt sich mit bisherigen Studien 8 11 12 13 .

Die Wahrscheinlichkeit einer erfolgreichen sialendoskopischen Steinextraktion war für Steine bis 4 mm sehr hoch (100 %), unabhängig von einer distalen oder proximalen Lokalisation. Diese Beobachtung war unabhängig von der betroffenen Speicheldrüsenlokalisation. Für distal gelegene Konkremente gestaltete sich die sialendoskopische Steinextraktion ab einer Steingröße von 5–6 mm schwieriger. Die Erfolgsrate betrug dann statt 100 % nur mehr etwa 50 %. Für proximal gelegene Konkremente war die Extraktion bereits ab 4–5 mm schwieriger. Die Erfolgsrate betrug hier 0 %. Die Gangdimension der Speicheldrüsen nimmt von distal nach proximal ab 18 . Dementsprechend limitieren insbesondere bei proximaler Steinlokalisation die eingeschränkte Öffnung des Steinfangkörbchens und inadäquate Konkrementmobilisierung die Extraktionsmöglichkeiten.

Eine Auswertung dieser Beobachtungen getrennt nach Speicheldrüse deutete ähnliche prognostische Einflussfaktoren (d. h. geringe Steingröße, distale Lokalisation) auf den Erfolg einer interventionellen Sialendoskopie an. Aufgrund der geringen Fallzahl der retrospektiven Studie erreichten diese Einflussfaktoren aber insbesondere für Patienten, die einer Sialendoskopie der Glandula parotis unterzogen wurden, kein Signifikanzniveau. Ebenfalls zeigte sich in der Glandula parotis für Konkremente klassifiziert als L _2a_ S _2_ D _0_ eine Erfolgsrate von 100 % ( Abb. 3 ). Jedoch gab es nur ein L _2a_ S _2_ D _0_ -Konkrement, dementsprechend könnte das auch dem Zufall zugeordnet werden.

Ein wesentlicher Nachteil unserer Studie lag in der fehlenden bzw. ungenügenden Zertrümmerung größerer Steine. Insbesondere blieb das Zertrümmerungsverfahren gerade für proximal gelegene größere Steine mit dem Spiralbohrer erfolglos. Ähnliche Untersuchungen berichteten, dass die Methode der Steinfragmentierung einen signifikanten Einflussfaktor auf die Erfolgsrate der interventionellen Sialendoskopie darstellt 9 . Auch wenn die im Rahmen der vorliegenden Studie verwendete Operationsausrüstung den aktuell geforderten Standards entspricht 19 , wurde rezent der zunehmende Stellenwert der Laserlithotripsie bei der interventionellen Sialendoskopie diskutiert 20 21 . Die Laserlithotripsie wurde im Rahmen dieser Studie nicht durchgeführt.

Noch ein möglicher Kritikpunkt unserer Studie ist die Durchführung aller Eingriffen in Vollnarkose. Mastrolonardo und Co-Autoren verglichen Ergebnisse von 98 Eingriffen in Vollnarkose mit Ergebnissen von 74 Eingriffen in Sedierung 22 . Laut Autoren reduzierte die Sedierung die Krankenhausaufenthaltsdauer um 141 Minuten, die Anästhesiedauer um 46 Minuten, die Operationsdauer um 24 Minuten, die Aufenthaltsdauer im Operationssaal um 43 Minuten und Erholungszeitdauer um 56 Minuten, wobei die chirurgischen Ergebnisse ähnlich zwischen den beiden Gruppen waren. Andere Studien berichteten ebenso über die Durchführung einer Sialendoskopie in Sedierung 7 8 23 , sogar bei Kindern für die juvenile rezidivierende Parotitis 24 25 . Coniglio und Co-Autoren berichteten noch über eine Reduktion der Kosten bei Durchführung des Eingriffs in Sedierung 26 . Ergänzend wurde in anderen Untersuchungen zu prognostischen Einflussfaktoren auf die Erfolgsrate bei interventioneller Sialendoskopie der Stellenwert von Steinvolumen, Orientierung des Konkrements im Speicheldrüsengang und Abstand der Konkremente zur Papille diskutiert. Diese Daten wurden im Rahmen der vorliegenden Studie nicht routinemäßig erhoben.

Die Sialendoskopie-Datenbank enthielt Informationen, die in mehreren Studien untersucht wurden 13 27 28 29 . Das Alter und die Geschlechterverteilung der Patienten stimmten mit dem Alter und der Geschlechterverteilung anderer Studien überein 13 27 28 29 . Auch die Anzahl der Eingriffe im untersuchten Zeitraum ähnelte der in anderen Studien 30 . Ähnlich wie Luers und Co-Autoren 31 konnten wir beobachten, dass die Dauer der Symptomatik in der Misserfolgsgruppe deutlich höher war als in der Erfolgsgruppe. Das Ergebnis war jedoch nicht signifikant. Die Eingriffe dauerten 30 Minuten länger als in anderen Studien berichtet 9 32 . Das könnte mit der geringeren Erfahrung der Operateure zusammenhängen 32 33 .

In unserer Studie beobachteten wir eine Erfolgsrate von 32 %. Diese zeigte sich zwar niedriger als in vergleichbaren Studien 9 12 13 27 30 32 34 . Diese Diskrepanz kann mit der Indikationsstellung und der Definition des Erfolgs zusammenhängen. Marchal und Co-Autoren berichteten über eine erfolgreiche Steinextraktion bei 90 % der Patienten mit einer Sialolithiasis der Glandula submandibularis. In dieser Studie betrug die durchschnittliche Steingröße 4,9 mm 9 . In unserer Studie betrug die Steingröße in der Glandula submandibularis im Mittel 8,9 mm und in der Glandula parotis 4,1 mm. Marchal und Dulguerov betonten, dass die obere Grenze für die erfolgreiche sialendoskopische Extraktion eines Steins 4 mm für die Glandula submandibularis und 3 mm für die Glandula parotis sei 8 . Dementsprechend könnte dadurch die deutlich niedrigere Erfolgsrate unserer Studie erklärt werden. Berücksichtigt man allerdings die Steingröße und Steinlokalisation als Einflussfaktoren auf die Erfolgsrate, waren die Erfolgsraten zwischen unserer und anderen Studien doch vergleichbar. Cox und Co-Autoren berichteten von einer Erfolgsrate der endoskopischen Steinextraktion von 51 % in der Glandula submandibularis und 47 % in der Glandula parotis in 85 Patienten, wenn man als Erfolg die reine sialendoskopische Steinextraktion (mit oder ohne Papillotomie) bezeichnet. Für Steine größer als 5 mm berichteten sie von einer Erfolgsrate von 22 % 13 . In unserer Studie beobachteten wir eine ähnliche Erfolgsrate für Steine größer als 5 mm (ca. 15 %). Diese Daten, insbesondere die Erfolgsrate je nach Steingröße, betonen die Wichtigkeit der Patientenselektion und der Indikationsstellung, vor allem bei fehlenden Möglichkeiten einer adäquaten Zertrümmerung.

Abschließend untersuchten wir die Korrelation bzw. die Übereinstimmung der Halssonografie und der Sialendoskopie in der Bestimmung der Steinanzahl. Die Durchführung der bildgebenden präoperativen Untersuchungen entsprach den aktuell geforderten Standards 19 . Die Anzahl der Steine in der Halssonografie korrelierte signifikant mit der Anzahl der Steine in der Sialendoskopie, jedoch war diese schwach. Die Bland-Altman-Methode deutete an, dass die Anzahl der Steine in der Sialendoskopie im Mittel 1,09-mal (± 1,19-mal) höher im Vergleich zu der Anzahl der Steine in der Halssonografie war ( Abb. 4 ). Yu und Co-Autoren berichteten ebenso von 32/561 Eingriffen mit einer höheren Anzahl von Steinen in der Sialendoskopie im Vergleich zur Anzahl der Steine in der Halssonografie 29 .

## Schlussfolgerung

Diese retrospektive Studie lieferte Daten über den Nutzen der LSD-Klassifikation bei der Prognoseeinschätzung einer interventionellen Sialendoskopie bei Sialolithiasis. Laut den vorliegenden Daten lag bei Steinen klassifiziert als L _1_ S _0_ D _0_ und L _2a_ S _0_ D _0_ die Erfolgsrate bei 50–100 %, was die der LSD-Klassifikation zugrunde liegenden Unsicherheiten verdeutlicht. Die LSD-Klassifikation zeigt damit deutliche Schwächen als möglicher prognostischer Einflussfaktor auf den Erfolg der interventionellen Sialendoskopie bei Sialolithiasis und erscheint daher für die Prognoseeinschätzung bei der Sialolithiasis im Allgemeinen nur bedingt geeignet.

Des Weiteren suggerieren die vorliegenden Daten, dass eine distale Steinlokalisation und kleine Steingröße in einem ansonsten unauffälligen Gangsystem günstige prognostische Einflussfaktoren auf den Erfolg bei einer interventionellen Sialendoskopie sein könnten. Aktuell stellen proximal gelegene, größere Steine insbesondere im Bereich der Glandula submandibularis eine Herausforderung dar. In solchen Fällen könnte ein alternatives Zertrümmerungsverfahren wie etwa die Laserlithotripsie den Extraktionserfolg verbessern. Laut der vorliegenden zusammengefassten Daten können Steine bis 4 mm verlässlich extrahiert werden, jedoch können Schwierigkeiten bei proximal gelegenen Konkrementen auch unter 4 mm auftreten. Bei Steinen über 4 mm scheint insbesondere die proximale Lokalisation prognostisch ungünstig zu sein. Des Weiteren sinkt die Erfolgsrate bei distal gelegenen Steinen zwischen 5 und 6 mm deutlich (ca. 50 %). Zukünftige Studien sollten anhand größerer Datenmengen die LSD-Klassifikation, das Volumen der Steine und deren Gangorientierung bzw. deren Abstand von der Papille untersuchen.
